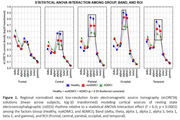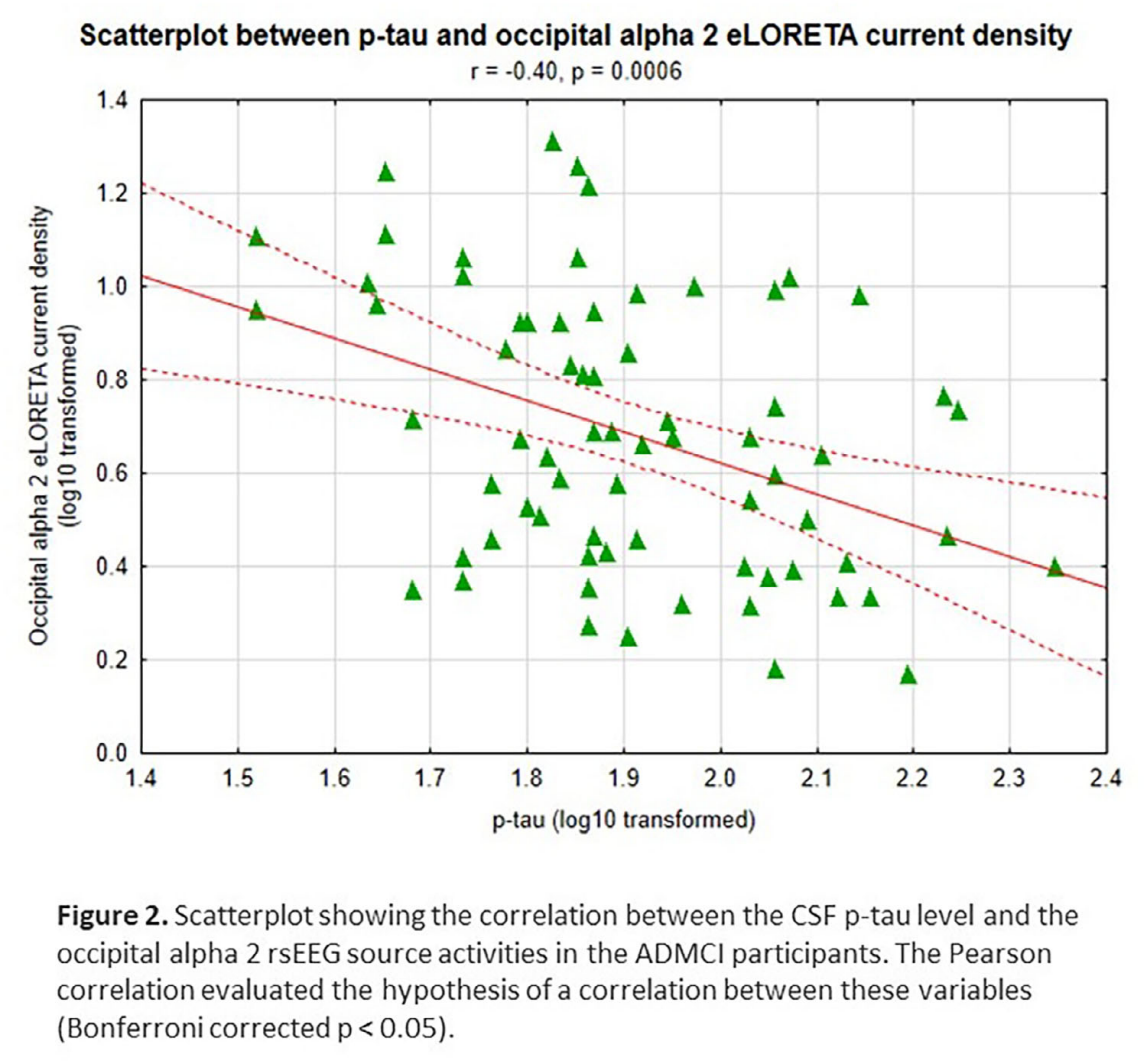# Relationship between Posterior Resting‐State Electroencephalographic Alpha Rhythms and Cerebrospinal Fluid Biomarkers of Neuropathology in Patients with Alzheimer’s Disease Mild Cognitive Impairment

**DOI:** 10.1002/alz.087262

**Published:** 2025-01-09

**Authors:** Claudio Del Percio, Roberta Lizio, Susanna Lopez, Giuseppe Noce, Dharmendra Jakhar, Matteo Carpi, Dario Arnaldi, Bahar Güntekin, Görsev Yener, Claudio Babiloni

**Affiliations:** ^1^ Sapienza University of Rome, Rome Italy; ^2^ IRCCS Synlab SDN, Naples Italy; ^3^ IRCCS Ospedale Policlinico San Martino, Genoa Italy; ^4^ University of Genoa, Genoa Italy; ^5^ Istanbul Medipol University, Istanbul Turkey; ^6^ Izmir University of Economics, Faculty of Medicine, Balçova, Izmir Turkey; ^7^ San Raffaele Cassino, Cassino Italy

## Abstract

**Background:**

Patients with mild cognitive impairment due to Alzheimer’s disease (ADMCI) are characterized by abnormalities in resting‐state electroencephalographic (rsEEG) rhythms as measures of brain neural synchronization dysfunction (Babiloni et al., PMID: 33860614). Here, we tested the two following hypotheses that those rsEEG abnormalities: (i) may be higher in ADMCI patients than in patients with MCI not due to AD (noADMCI); and (ii) may be related to the AD diagnostic amyloid‐tau biomarkers derived from cerebrospinal fluid (CSF).

**Method:**

Datasets in 70 ADMCI patients, 45 patients noADMCI, and 45 normal elderly (Healthy) participants originated from a Eurasian archive. The eyes‐closed rsEEG rhythms were investigated at individual delta, theta, and alpha frequency bands and fixed beta and gamma bands. The eLORETA freeware estimated rsEEG cortical sources.

**Result:**

Statistical results showed: (i) compared with the Healthy group, the posterior alpha rsEEG source activities were more reduced in the ADMCI group than in the noADMCI group (p < 0.001; Figure 1); (ii) in the ADMCI group, a strong negative correlation between the CSF phospho‐tau levels and the occipital alpha rsEEG source activities was observed (r = 0.4, p < 0.001; Figure 2). Interestingly, in the ADMCI group, no correlation between the CSF and magnetic resonance imaging (MRI; neurodegeneration) variables was observed.

**Conclusion:**

In ADMCI patients, the abnormalities of cortical neural synchronization mechanisms underpinning brain arousal and vigilance are related to the AD diagnostic phospho‐tau biomarkers and precede a similar relationship between AD amyloid‐tau and neurodegeneration biomarkers. Remarkably, the amyloid‐tau‐neurodegeneration (ATN) AD model may be enriched with those pathophysiological ‘P’ biomarkers as revealed by rsEEG rhythms.